# A Randomized, Placebo-Controlled Trial of Omega-3 Fatty Acids for Inhibition of Supraventricular Arrhythmias After Cardiac Surgery: The FISH Trial

**DOI:** 10.1161/JAHA.111.000547

**Published:** 2012-06-22

**Authors:** Chirag M. Sandesara, Mina K. Chung, David R. Van Wagoner, Thomas A. Barringer, Keith Allen, Hassan M. Ismail, Bridget Zimmerman, Brian Olshansky

**Affiliations:** From Virginia Cardiovascular Associates, Manassas, VA (C.M.S.); The Cleveland Clinic, Cleveland, OH (M.K.C., D.R.V.W.); Presbyterian Heart and Wellness, Charlotte, NC (T.A.B.); Mid America Heart and Vascular Institute, Kansas City, MO (K.A.); Detroit Medical Center Cardiovascular Institute, Wayne State University, Detroit, MI (H.M.I.); University of Iowa Hospitals and Clinics, Iowa City, IA (B.Z., B.O.)

**Keywords:** fatty acids, coronary artery bypass graft surgery, atrial fibrillation

## Abstract

**Background:**

Omega-3 polyunsaturated fatty acids (n3-PUFAs) might have antiarrhythmic properties, but data conflict on whether n3-PUFAs reduce rates of atrial fibrillation (AF) after coronary artery bypass graft surgery (CABG). We hypothesized that n3-PUFAs would reduce post-CABG AF, and we tested this hypothesis in a well-powered, randomized, double-blind, placebo-controlled, multicenter clinical trial.

**Methods and Results:**

Patients undergoing CABG were randomized to pharmaceutical-grade n3-PUFAs 2 g orally twice daily (minimum of 6 g) or a matched placebo ≥24 hours before surgery. Gas chromatography was used to assess plasma fatty acid composition of samples collected on the day of screening, day of surgery, and postoperative day 4. Treatment continued either until the primary end point, clinically significant AF requiring treatment, occurred or for a maximum of 2 weeks after surgery. Two hundred sixty patients were enrolled and randomized. Before surgery, n3-PUFA dosing increased plasma n3-PUFA levels from 2.9% to 4% and reduced the n6:n3-PUFA ratio from 9.1 to 6.4 (both *P*<0.001). Similar changes were noted on postoperative day 4. There were no lipid changes in the placebo group. The rate of post-CABG AF was similar in both groups (30% n3-PUFAs versus 33% placebo, *P*=0.67). The post-CABG AF odds ratio for n3-PUFAs relative to placebo was 0.89 (95% confidence interval 0.52–1.53). There were no differences in any secondary end points.

**Conclusions:**

Oral n3-PUFA supplementation begun 2 days before CABG did not reduce AF or other complications after surgery.

**Clinical Trial Registration:**

url: http://www.clinicaltrials.gov Unique identifier: NCT00446966. **(*J Am Heart Assoc*. 2012;1:e000547 doi: 10.1161/JAHA.111.000547.)**

## Introduction

A trial fibrillation (AF) complicates management after coronary artery bypass graft surgery (CABG).^1–3^ The incidence of postoperative AF (POAF) is 10% to 50% and is higher in elderly patients.^4,5^ POAF usually occurs 2 to 5 days after CABG, may increase hospital costs and length of stay, and is associated with an increased risk of morbidity, mortality, stroke, ventricular arrhythmias, need for pacemaker therapy, and use of potentially proarrhythmic drugs.^6–12^ Inflammatory and autonomic mechanisms are thought to underlie POAF.^12^

Omega-3 polyunsaturated fatty acids (n3-PUFAs) have antiarrhythmic and antiinflammatory properties, augment vagal tone, and reduce interstitial fibrosis.^13^ Arachidonic acid, an n6-PUFA released as a result of ischemia or inflammation, can slow atrial conduction and promote POAF.^14^ Arachidonic acid metabolites (especially leukotriene B_4_) are implicated in recruiting neutrophils into injured myocardium.^15^ Neutrophil infiltration is associated with heterogeneous conduction and AF inducibility in the postoperative setting.^16^ Interventions that increase atrial n3-PUFA content and lower the n6:n3 ratio might be expected to decrease POAF. In a canine preclinical study, a 3-week period of pretreatment with fish oil was associated with slower postoperative heart rate, lower plasma and atrial arachidonic acid abundance, less neutrophil infiltration, and enhanced heart rate variability. AF was inducible with atrial burst pacing in 4 of 6 control animals but in none (0 of 7) of the fish oil–treated animals.^17^

Population-based observational studies show an association between fish intake and the incidence of AF, but results conflict.^18–20^ Kowey et al ^21^ recently reported a randomized, double-blind, placebo-controlled trial involving 663 patients with either paroxysmal or persistent AF. Eight grams per day of n3-PUFA or placebo were given for 7 days, and then 4 g/d were given thereafter for 24 weeks. In that study, fish oil treatment had no effect on the recurrence of symptomatic AF in the paroxysmal, persistent, or combined groups (all *P* > 0.05).^21^

AF in the nonsurgical population may be mechanistically distinct from AF after cardiac surgery, with inflammatory mechanisms likely having a greater role in the postsurgical patient. With regard to prevention of POAF with supplemental n3-PUFA, results of the first clinical trial were encouraging,^22^ but subsequent clinical trials have yielded conflicting results.^23–26^ On the basis of the initial report,^22^ we hypothesized that n3-PUFAs would reduce the occurrence of POAF. The purpose of the present study was to perform a randomized, placebo-controlled, multicenter trial to determine if oral supplementation with n3-PUFAs reduces the incidence of post-CABG AF.

## Methods

This was a randomized, double-blind, parallel-group, multicenter trial that compared treatment with a pharmaceutical-grade n3-PUFA to a matched corn oil (n6-PUFA) placebo. The protocol for this investigator-initiated and investigator-designed study was approved by each of the centers’ Institutional Review Boards, and all patients gave informed consent. The study was registered with http://www.ClinicalTrials.org (NCT00446966) and was funded by Reliant Pharmaceuticals, Liberty Corner, NJ, and GlaxoSmithKline, Research Triangle Park, NC. An independent clinical research organization (Quality Data Services, Inc, King of Prussia, PA) was responsible for data management, randomization, and monitoring.

### Patient Population

Patients were screened, interviewed, and enrolled in presurgical cardiothoracic clinics. Patients were identified ≥2 days before scheduled CABG. Patients who were 18 to 85 years old were included if they were undergoing elective CABG with or without concomitant valve surgery. Patients were excluded if they had any of the following: emergency CABG, unstable angina or heart failure, persistent AF, AF at the time of screening, planned Maze procedure or pulmonary vein isolation, warfarin administration 48 hours before surgery, use of Vaughan-Williams Class I or III antiarrhythmic drugs within 5 elimination half-lives of the drug (or within 2 months for amiodarone), a pacemaker or implantable cardioverter-defibrillator (because of the use of AF suppression algorithms by the device), n3-PUFA supplementation at the time of screening, pregnancy, or inability to provide consent.

### Study Treatment

Patients were randomized to omega-3-acid ethyl esters (Omacor/Lovaza) or a matching corn oil placebo, both supplied by Reliant Pharmaceuticals, Liberty Corner, NJ, and GlaxoSmithKline, Research Triangle Park, NC. Each 1-g capsule of n3-PUFA contained ≥900 mg of omega-3 fatty acid ethyl esters (465 mg eicosapentaenoic acid [EPA] and 375 mg docosahexaenoic acid [DHA]). The presurgical loading dose was 2 g orally twice daily for a minimum of 6 g, with no maximum dose. After surgery, treatment continued with 2 g/d until the primary end point, AF, was reached or 14 days had passed (whichever occurred first). The study drug was reinitiated after extubation.

### Data Collection

Plasma samples for n3-PUFA analyses were obtained on day of screening, on day of surgery, and on postoperative day (POD) 4. Fish consumption was self-reported in the following categories: >2 times per week, 1 to 2 times per week, 1 to 3 times per month, <1 time per month, and none. Patients had daily 12-lead electrocardiograms and continuous telemetry monitoring while hospitalized. Rehospitalization and treatment for AF were monitored. All patients were contacted by telephone on PODs 14 and 30 to assess for adverse events and to determine if POAF had been documented during any hospitalization or office visit according to patient self-report. A Data and Safety Monitoring Board monitored study progress, patient safety, and adverse events.

### Study End Points

The primary end point was AF or atrial flutter, defined as any episode of AF or flutter that in the opinion of the treating physician required additional pharmacological treatment (eg, digoxin, additional β-adrenergic blockers, calcium channel blockers, class I or III antiarrhythmic drugs), electrical or pharmacological cardioversion, or atrial overdrive pacing. Follow-up continued until the primary end point was met or 14 days had passed, whichever occurred first. Prespecified secondary end points are listed in Table 3. Data on time in POAF and number of episodes also were collected. All end points were independently adjudicated by 2 blinded cardiologists.

**Table 3.** Primary and Secondary Post-CABG Outcomes

Outcome Variable	n3-PUFA (n=120)	Placebo (n=123)	P*
AF/flutter requiring therapy	36 (30%)	40 (33%)	0.67
Post-CABG length of stay, d, median (25th–75th percentile)	6 (5–8)	5 (4–7)	0.27†
Congestive heart failure	3 (2%)	2 (2%)	0.68
Sustained ventricular arrhythmias	1 (1%)	2 (2%)	1.0
Myocardial infarction	1 (1%)	1 (1%)	1.0
Bleeding requiring reoperation or transfusion	22 (18%)	15 (12%)	0.18‡
Infection	14 (12%)	13 (11%)	0.79‡
Renal failure	4 (3%)	4 (3%)	1.0
Respiratory failure	2 (2%)	3 (2%)	1.0
Stroke or transient ischemic attack	3 (2%)	3 (2%)	1.0
Rehospitalization for AF	1 (1%)	2 (2%)	1.0
Readmission to intensive care unit	7 (6%)	9 (7%)	0.64‡
Death within 30 d	0 (0%)	0 (0%)	1.0

Values are given as n (%) of patients unless otherwise noted.

* Fisher exact test, except where indicated by † or ‡.

† Wilcoxon rank-sum test.

‡ Pearson χ^2^ test.

### Sample Size and Power

Sample size for the primary end point was computed on the basis of results from Calò et al^22^ in which the incidence of AF was 15.2% in the treatment group versus 33.3% in the placebo group. Both Calò et al^22^ and Saravanan et al^23^ based their calculations on the assumption of a relative risk reduction of 54% by n3-PUFAs. With 116 patients per treatment group, and with Pearson χ^2^ test at the 0.05 significance level, the present study was powered to detect a difference of ≥18.1% (corresponding to a 54% reduction) in the incidence of POAF for the n3-PUFA group relative to the placebo group (ie, 15.2% versus 33.3%), with 90% power. To account for possible patient withdrawals, sample size was increased by 12%, for a total enrollment of 260 patients.

### Plasma Fatty Acid Analysis

Plasma was collected on day of screening, day of surgery, and POD 4 to evaluate its fatty acid composition. Five milliliters of whole venous blood was collected in a purple-top tube and centrifuged at ≈2000*g* for 10 minutes. Plasma was stored at −80°C until analysis (Lipid Technologies, Austin, MN). Lipid composition was determined by capillary gas chromatography.^26^

### Statistical Analysis

Data were analyzed on an intention-to-treat basis, where all patients who were randomized and had CABG surgery were included in the analysis. The Pearson χ^2^ test for equality of proportions was used to compare the primary end point of POAF between the n3-PUFA and placebo groups. The categorical secondary end points and categorical demographic, clinical, and CABG variables were compared by either Pearson χ^2^ test or Fisher exact test. The Wilcoxon rank-sum test was used to compare the duration of POAF, the secondary outcome variable of hospital length of stay, and duration of POAF. Wilcoxon rank-sum test was also used to compare fish consumption and CABG variables, including the number of valves replaced. The 2-sample Student *t* test was used to compare mean heart rate, and the log-rank test was used for the comparison of time to POAF between the 2 treatment arms. Linear mixed-model analysis for repeated measures was used to compare mean plasma levels of n3-PUFAs and n6:n3-PUFA ratios measured at screening, surgery, and POD 4 between the n3-PUFA and placebo groups. All statistical analyses were performed in SAS 9.2 (Cary, NC).

## Results

**Figure 1.**
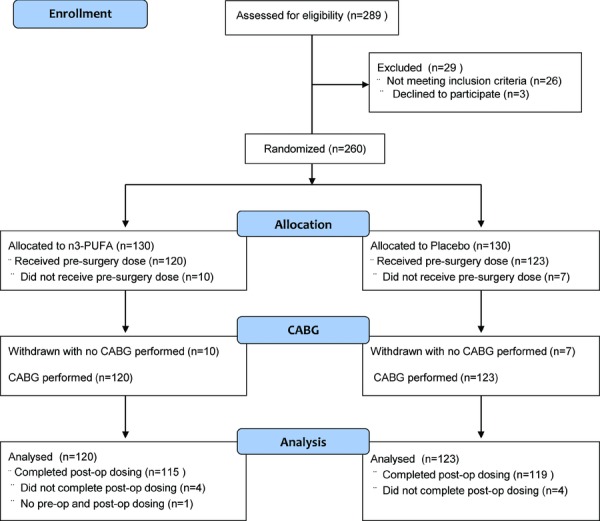
Flow chart.

From February 2007 until December 2009, 260 patients were enrolled (130 in each arm). Seventeen patients(10 randomized to n3-PUFA and 7 randomized to placebo) did not undergo CABG, and their data were excluded. The remaining 243 patients (120 in the n3-PUFA versus 123 in the placebo arm) were followed up until study completion (Figure 1). There were no baseline differences between groups. Most patients were male (81%), with an average age of 62.7 years (Table 1). The median dose (25th–75th percentile) of presurgical study drug taken was 8 (6–10) g for n3-PUFA and 6 (6–10) g for placebo (*P*=0.58). The median duration (25th–75th percentile) of preoperative study drug administration was 2.5 (2–3) days for n3-PUFA and 2 (2–3) days for placebo (*P*=0.47). Baseline fish consumption did not differ between groups (*P*=0.35). No patient required a nasogastric tube for study drug administration during the postoperative period. There were no differences in the number of grafts placed (venous or arterial), number and location of valves replaced or repaired, or number of off-pump CABGs performed between groups (all *P* > 0.05) (Table 2).

**Table 1.** Baseline Demographic and Clinical Data

Variable	n3-PUFA (n=120)	Placebo (n=123)
Sex, male	94 (78%)	102 (83%)
Age, y, mean±SD	63.4±9.5	62.0±11.4
Race		
White	109 (91%)	116 (94%)
African American	10 (8%)	6 (5%)
Other	1 (1%)	1 (1%)
Myocardial infarction	47 (39%)	54 (44%)
Congestive heart failure	12 (10%)	13 (11%)
Left ventricular ejection fraction, %, mean±SD	(n=68) 52.0±15.4	(n=75) 53.4±13.5
Left atrial size, cm, mean±SD	(n=56) 3.8±0.8	(n=60) 4.0±0.8
Aortic stenosis	21 (18%)	21 (17%)
Aortic regurgitation	4 (3%)	8 (6%)
Mitral stenosis	2 (2%)	0 (0%)
Mitral regurgitation	17 (14%)	22 (18%)
Hypertension	107 (89%)	108 (88%)
Hyperlipidemia	97 (81%)	89 (72%)
Diabetes	45 (38%)	43 (35%)
Chronic renal failure	4 (3%)	1 (1%)
Hypothyroidism	8 (7%)	8 (6%)
History of pulmonary embolism	2 (2%)	3 (2%)
Obstructive lung disease	21 (18%)	14 (11%)
Drugs		
β-Adrenergic blocker	97 (81%)	97 (79%)
Angiotensin-converting enzyme inhibitor	65 (54%)	58 (47%)
Statin	90 (75%)	90 (73%)
Fish consumption		
>2 times per week	12 (10%)	12 (10%)
1–2 times per week	34 (28%)	33 (28%)
1–3 times per month	56 (47%)	54 (44%)
<1 time per month	12 (10%)	13 (11%)
None	6 (5%)	11 (9%)

Values are given as n (%) of patients unless otherwise noted.

**Table 2.** CABG and Valve Replacement/Repair Data

Variable	n3-PUFA (n=120)	Placebo (n=123)	*P*
Number of saphenous vein grafts			0.80*
0	9 (8%)	12 (10%)	
1	33 (28%)	31 (25%)	
2	47 (39%)	51 (41%)	
3	25 (21%)	21 (17%)	
4–5	6 (5%)	8 (6%)	
Number of internal mammary artery grafts			0.15*
0	15 (12%)	12 (10%)	
1	99 (82%)	98 (80%)	
2	5 (4%)	12 (10%)	
3	1 (1%)	1 (1%)	
Valve replacement/repair			0.76†
Aorta	14 (12%)	11 (9%)	
Mitral	1 (1%)	1 (1%)	
Tricuspid	0 (0%)	1 (1%)	
No valve replacement/repair	105 (88%)	110 (89%)	
Off-pump bypass	28 (23%)	33 (27%)	0.53‡

Values are given as n (%) of patients.

* Wilcoxon rank-sum exact test.

† Fisher exact test for r×c table.

‡ Pearson χ^2^ test.

Overall incidence of POAF was 31% (76 of 243). The incidence of POAF did not differ between n3-PUFA and placebo groups (*P*=0.67). POAF occurred in 36 of 120 patients (30%; 95% confidence interval [CI] 22%–38%) in the n3-PUFA group and in 40 of 123 patients (33%; 95% CI 24%–41%) in the placebo group, with an odds ratio of POAF for n3-PUFAs relative to placebo of 0.89 (95% CI 0.52–1.53). Two patients had POAF after hospital discharge (one in each group). AF duration was recorded for 67 (n=29 for n3-PUFA and n=38 for placebo group) of the 76 patients with POAF. The median (25th–75th percentile) duration of AF was 7.9 (2.4–30.8) hours for the n3-PUFA group and 10.6 (3.0–34.7) hours for the placebo group (*P*=0.68). POAF was treated by antiarrhythmic drugs (88%), cardioversion (3%), and other methods (9%). Antiarrhythmic drugs were given to 67 patients, with 67% of these receiving amiodarone. There was no significant difference in the postoperative hospital length of stay between groups (*P*=0.27), with a median (25th–75th percentile) length of stay of 6 (5–8) days in the n3-PUFA group versus 5 (4–7) days in the placebo group. Other postoperative complications, including death, were similar between groups (all *P* > 0.05; Table 3).

**Figure 2.**
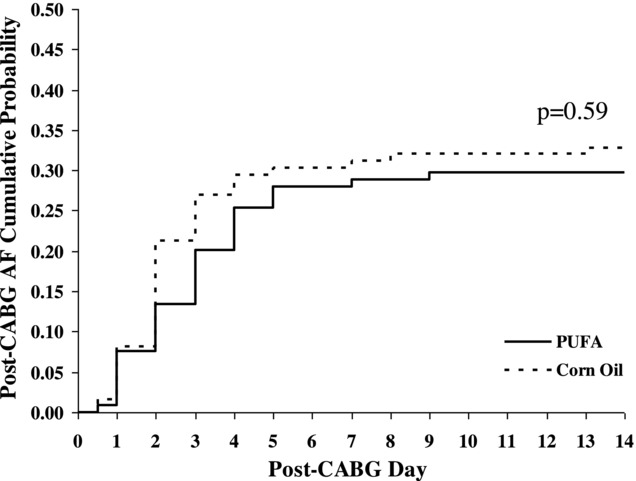
Kaplan-Meier curve: time to AF after CABG.

Kaplan-Meier analysis of time to AF demonstrated no difference between the n3-PUFA and placebo groups (log-rank test *P*=0.59; Figure 2). By POD day 7, 29% (95% CI 22%–39%) had AF in the n3-PUFA group, versus 31% (95% CI 24%–40%) in the placebo group.

No adjustment for confounders or covariates was needed for the tests comparing primary and secondary end points between the n3-PUFA group and the placebo group because no significant differences in demographic, clinical, or CABG variables were found between the 2 groups (see Table 1 and Table 2).

Linear mixed-model analysis was used to compare the plasma n6:n3-PUFA ratio and plasma EPA+DHA levels measured at 3 time points (screening, surgery, and POD 4) between the n3-PUFA group and the placebo group. This showed a significant treatment×time interaction effect for both plasma variables (both *P* > 0.0001). There was a significant change in mean plasma EPA+DHA levels over time in each of the 2 groups (*P*<0.0001 for n3-PUFA group; *P*=0.0018 for placebo group), with the n3-PUFA group having a significantly greater change from screening to POD 4 than that of the placebo group. This was similarly seen for n6:n3-PUFA ratio (time effect *P*<0.0001 for n3-PUFA group; *P*=0.0035 for placebo group). At the time of screening, there were no differences in the plasma n6:n3-PUFA ratio or the plasma EPA+DHA levels between groups (Table 4). On both day of surgery and POD 4, mean plasma EPA+DHA levels were significantly higher in the n3-PUFA group than in the placebo group (both *P*<0.001). As a consequence, the n6:n3-PUFA ratio in patients receiving n3-PUFAs was lower than in those receiving placebo on the day of surgery and at POD 4 (both *P*<0.001). There were no significant differences in plasma fatty acid levels in patients who had POAF compared to those who did not. The mean n6:n3-PUFA ratio was 7.7 (95% CI 6.9–8.6) in those with POAF, compared to 7.6 (95% CI 7.1–8.0) in those who did not have POAF (*P*=0.76).

**Table 4.** Comparison of Plasma Fatty Acid Composition Between Treatment Groups on Day of Screening, Day of Surgery, and POD 4

Group and Time	n3-PUFA (n=100)	Placebo (n=101)	*P*
n6:n3			
Screening	9.10 (8.54–9.70)	9.11 (8.65–9.61)	>0.99
Surgery	6.44 (5.99–6.93)	9.31 (8.84–9.81)	<0.001
POD 4	6.18 (5.89–6.48)	8.64 (8.23–9.08)	<0.001
EPA+DHA			
Screening	2.89 (2.66–3.11)	2.88 (2.68–3.09)	>0.99
Surgery	4.35 (4.00–4.70)	2.78 (2.61–2.95)	<0.001
POD 4	4.27 (4.02–4.51)	3.03 (2.85–3.21)	<0.001

Test for change over time in n6:n3: *P*<0.0001 in n3-PUFA and *P*=0.0018 in placebo.

Test for change over time in EPA+DHA: *P*<0.0001 in n3-PUFA and *P*=0.0035 in placebo.

n6:n3 indicates the ratio of n6:n3-PUFA in plasma phospholipids; EPA+DHA, fractional contribution (%) of EPA and DHA to plasma phospholipids.

All values reflect the mean (95% CI).

Data from the electrocardiogram and telemetry monitoring were recorded and analyzed for trends. Heart rate (mean ± standard deviation [SD]) was 82.2±10.6 bpm in patients who developed POAF and 85.9±11.1 bpm in those without POAF. Subjects who developed POAF had a significantly lower heart rate than those who maintained normal sinus rhythm (*t* test *P*=0.016). Mean heart rate for patients with POAF was 81.4±11.8 bpm in the n3-PUFA group versus 82.9±9.5 bpm in the control group (*t* test *P*=0.56). For all patients receiving n3-PUFAs (mean heart rate 84.5±11.0 bpm) compared to control patients (mean heart rate 85.0±10.9 bpm), there was no significant difference in the mean heart rate (*t* test *P*=0.72).

## Discussion

This double-blind, randomized, placebo-controlled, multicenter trial provided no evidence to support the hypothesis that oral n-3 PUFA supplementation begun 2 days before CABG reduces the incidence of postoperative AF.

In the first study on this topic, Calò et al^22^ performed a randomized, open-label trial of 160 patients undergoing elective isolated CABG. A dose of 2 g/d of n3-PUFA was started a mean of 5 days before surgery. POAF occurred in 27 patients in the placebo group (33.3%) versus 12 patients in the n3-PUFA group (15.2%) (*P*=0.013). The n3-PUFA group was hospitalized for fewer PODs than were controls (7.3±2.1 versus 8.2±2.6 days, *P*=0.017). This was an open-label study, and no data were provided on plasma lipid levels.

Saravanan et al^23^ published a randomized, double-blind, placebo-controlled, single-center trial in 108 patients, reporting no difference in POAF when comparing 2 g/d of n3-PUFA to placebo (95% CI −6% to 30%, *P*=0.28), with oral treatment beginning ≥5 days before surgery.^23^ Heidarsdottir et al^24^ performed a similar single-center, randomized, double-blind, placebo-controlled trial in 168 patients, using a slightly higher perioperative oral dose of 2.2 g of n3-PUFA beginning 5 to 7 days before surgery. This study also demonstrated no reduction of POAF (54.2% versus 54.1% comparing n3-PUFA to placebo, *P*=0.99).^24^

In contrast, Heidt et al^25^ reported that large intravenous doses (100 mg/kg per day) of n3-PUFAs begun on admission to the hospital for elective CABG reduced POAF. AF occurred in 15 patients (30.6%) in the control group and in 9 patients (17.3%) in the n3-PUFA group after CABG (*P*<0.05). The total dose of n3-PUFA used in this intravenous study was much higher than in the oral delivery studies. No data were reported on the safety or adverse effects of the regimen or on the plasma levels of n3-PUFA achieved. Thus, it is not possible to compare plasma levels in the Heidt et al^25^ study with our data.

The most recent study assessing the utility of n3-PUFA to reduce POAF again demonstrated no benefit in a CABG population with valve replacement surgery. Two hundred patients were randomized to receive fish oil (providing 4.6 g/d of n3-PUFA) or a placebo. There were no differences between groups with regard to the incidence of POAF (odds ratio 0.63, 95% CI 0.35–1.11), but patients taking n3-PUFA had a statistically shorter length of stay in the intensive care unit.^26^

Mariscalco and colleagues^27^ examined the role of n3-PUFAs in both early and late prevention of AF after CABG. They showed a significant reduction of POAF in the hospitalized group taking fish oil but no benefit when patients were in rehabilitation. Early AF occurred in 31.0% of the patients with preoperative PUFAs, compared with 47.3% of those without preoperative PUFAs (*P*=.006).^27^

All of the aforementioned CABG studies were single-center trials limited to 80% power studying a smaller population of patients. The present study differs from prior reports in design and in pre-CABG drug treatment duration. The present analysis is the first multicenter study in the United States to evaluate whether oral n3-PUFAs reduce the incidence of POAF. This study used a randomized, placebo-controlled trial design with 90% power to detect a 54% reduction in POAF. The previous orally administered n3-PUFA studies prescribed the study drug ≥5 days before surgery. Although our patient population received n3-PUFAs for a shorter duration (median 2.5 days), we were able to administer a minimum of 6 g (median dose 8 g) before surgery. This resulted in a significant augmentation of plasma n3-PUFA levels.

The conflicting data with regard to the impact of n3-PUFAs on the incidence of POAF after surgery were further evaluated in a recent meta-analysis that pooled data from 10 randomized, controlled, clinical trials (1955 patients), including the present study, and reported no demonstrable benefit of n3-PUFAs.^28^

Our results are concordant with 2 prior oral placebo-controlled studies supporting the conclusion that short-term perioperative oral n3-PUFA supplementation does not reduce the incidence of POAF despite augmentation of n3-PUFA plasma levels. The study drug dosing regimen was well tolerated without significant differences in adverse event rates compared to placebo.

The present study documented increased plasma PUFA levels with n3-PUFA administration, but the increase in fatty acid levels did not reduce the incidence of POAF. With an oral dosing regimen, it is unclear whether a higher n3-PUFA dose or longer pretreatment would be able to achieve adequate levels to cause a reduction in events. In an animal model, a 12-week n3-PUFA pretreatment reduced susceptibility to experimentally induced AF, but very high n3-PUFA tissue levels were achieved in the actively treated group (15.4%) compared to the control group (3.1%).^29^ Whether lower doses of n3-PUFA can achieve similar protection against AF in animal models or patients is unknown. In the present study, treatment increased the abundance of EPA and DHA in the plasma total lipid pool from 2.9% to ≈4% (Table 3). Longer duration of pretreatment with n3-PUFA has been shown to have a significant effect on atrial lipid composition. A pharmacodynamic study demonstrated that 2 weeks are required to double atrial n3-phospholipid content by using a dietary supplement.^30^ Thus, it is possible that higher tissue levels of n3-PUFA than we achieved could reduce the incidence of POAF. Further dose–response studies must be performed before another randomized clinical trial is done.

Prior research indicates changes in autonomic tone may be responsible for the genesis of AF in the muscular sleeves of pulmonary veins. A primary increase in sympathetic tone followed by a marked change toward vagal predominance might occur immediately before AF onset.^31^ n3-PUFAs also have demonstrated the ability to augment vagal tone when heart rate variability is examined.^32^ There might be a potential proarrhythmic effect of n3-PUFAs due to this, but our data do not support any vagally influenced effect based on heart rate changes alone. Certainly, further research is required to corroborate these relationships. The beneficial effect of n3-PUFAs on autonomic tone in humans, specifically an increase in vagal tone, might still reduce the risk of arrhythmias.

It might not be possible to achieve adequate n3-PUFA tissue levels with the oral dosing regimen used in the present study in pre-CABG patients to reduce the incidence of POAF. Still, this study does not exclude the possible antiarrhythmic benefit of n3-PUFAs that might be found in future trials that administer larger pre-CABG oral doses, combine n3-PUFAs with other antiarrhythmic agents, or administer n3-PUFAs via intravenous delivery.

The definition of POAF merits attention. Saravanan et al defined their primary end point as any episode of AF lasting >30 seconds on continuous monitoring.^23^ This might explain the higher rate of POAF in their study (43% in the placebo group and 56% in the n3-PUFA group). Similar to our study, others have reported an incidence of POAF close to 30%.^1,2^ The definition in our study, any AF or flutter requiring pharmacological or nonpharmacological intervention, was designed to detect clinically significant end points and was less likely to detect asymptomatic, brief episodes that did not require treatment.

POAF remains a major public health problem in that it increases hospital costs, length of hospital stay, and risk of death.^5,6,11^ Studies are being performed to further understand the mechanism of n3-PUFAs, the dose and duration of therapy needed, and how n3-PUFA therapy could be better applied to reduce the incidence of POAF.^33^

### Limitations

The primary end point of this study was the occurrence of POAF events that required therapy. Events that were not clinically significant were not treated and therefore were not considered to have met the primary end point. This introduces some variability in the treatment of POAF, given that institutional cultures vary, and some clinically relevant POAF events might not have been treated, depending on physicians’ discretion. Patients were discharged home and followed up 14 days after surgery. Home monitoring was not performed, and the incidence of posthospitalization AF was limited to reported episodes. Primary end-point assessment after hospitalization was determined by telephone interview of the study patients. Other episodes of POAF might have occurred unbeknownst to the patient and investigator.

The preoperative oral dosing regimen might not have achieved adequate levels of n3-PUFA. The adequate pre-CABG dose of n3-PUFA and duration of therapy to reduce POAF remain unclear. That these crucial pieces of information are still unknown might limit our ability to reach the threshold needed to affect myocyte cellular membranes and ion channels. However, our results are concordant with other randomized placebo-controlled studies of oral n3-PUFA.^21,23^

### Conclusions

This double-blind, randomized, placebo-controlled, multicenter clinical trial failed to provide evidence that oral n-3 PUFA supplementation begun 2 days before CABG reduces postoperative AF.
